# Frequency of Immediate Neonatal Complications (Hypoglycemia and Neonatal Jaundice) in Late Preterm and Term Neonates

**DOI:** 10.7759/cureus.12512

**Published:** 2021-01-05

**Authors:** Muhammad Salman, Heeranand Rathore, Shabina Arif, Rashid Ali, Ausaf A Khan, Muhammad Nasir

**Affiliations:** 1 Pediatrics and Child Health, The Aga Khan University, Karachi, PAK; 2 Pediatrics and Child Health, The Aga Khan University Hospital, Karachi, PAK; 3 Pulmonary and Critical Care Medicine, South City Hospital, Karachi, PAK; 4 Anesthesiology, The Aga Khan University, Karachi, PAK; 5 Anesthesiology and Pain Medicine, South City Hospital, Karachi, PAK; 6 Critical Care Medicine, South City Hospital, Karachi, PAK

**Keywords:** term neonates, late preterm neonates, jaundice, hypoglycemia, infant

## Abstract

Background

Evidence suggests that neonates born at 34-36 weeks should not be considered full-term neonates, given the magnitude of morbidities they experience compared with term infants. Neonates born at 34 to 36 weeks are at increased risk for early illness such as hypoglycemia and hyperbilirubinemia compared to term infants.

Objective

This study's objective was to determine the frequency of immediate neonatal complications (hypoglycemia and neonatal jaundice) in late preterm and term neonates.

Subjects and methods

A serial descriptive case study was conducted at the private tertiary care hospital. Random samplings were taken, and the sample size was calculated on Epi Info software (Centers for Disease Control and Prevention, Atlanta, GA). All the eligible samples were taken into confidence following approval by the College of Physicians and Surgeons Pakistan's institutional review board. A structured questionnaire was used in which demographic information of the patient was collected, and all neonates were closely observed for early targeted morbidities (hypoglycemia, hyperbilirubinemia)

Results

A total of 215 neonates were born during the study period, of whom 108 (50.2%) were term babies and 107 (49.8%) late preterm babies. There were 122 (56.7%) male infants and 93 (43.3%) female infants. Jaundice was observed in 6.5% (n=7) of term neonates and 22.4% (n=24) of late preterm neonates (p<0.0). Similarly, hypoglycemia was observed in only 4.6% (n=5) of term neonates and 15.9% (n=17) of late preterm neonates (p<0.01).

Conclusion

There is a significant association between gestational age and immediate neonatal complications of jaundice and hypoglycemia. Compared with term neonates, late preterm neonates are at a higher risk of neonatal jaundice and hypoglycemia. Gender and mode of delivery did not correlate to complications rate.

## Introduction

Late preterm births account for 74% of premature births and 10% of all live births [1]. The United States has seen an increase in premature births, from 9.4% to 12.7% [2]. The increase has been attributed to infertility treatments, increasing maternal comorbidity, obesity, and multiple gestations, along with increasing maternal age. The dramatic rise in the late preterm births (i.e., born at 34 weeks to 36 weeks plus six days) has contributed to the increase in preterm births. Immediate complications such as hypothermia, hypoglycemia, jaundice, sepsis, and respiratory distress have been documented in term late preterm neonates. Wang et al. reported that these clinical outcomes differed significantly between term and late preterm neonates [3]. Hypoglycemia was documented in 15.6% in late preterm neonates and 5.3% in term neonates (p=0.028) [3]. Hypoglycemia in neonates adversely affects brain development if untreated properly [4].

Neonatal jaundice is also a well-known immediate complication in newborn babies. Approximately 55% of late preterm and 24.8% of term neonates develop jaundice (p<0.001) [5]. Most neonates develop jaundice without any underlying cause (i.e., physiological jaundice) due to liver immaturity at birth, while others may have added risk factors [6].

To our knowledge, there is no study done in Pakistan on the frequency of hypoglycemia and neonatal jaundice in late preterm and term neonates. Therefore, the goal of this study was to determine the frequency of immediate neonatal complications (e.g., hypoglycemia and neonatal jaundice) in neonates born in Pakistan at 34 to 40 weeks plus six days compared to those born late preterm (i.e., 34 to 36 weeks plus six days) and term (i.e., 37 weeks to 40 weeks plus six days).

## Materials and methods

We conducted a descriptive, serial case study at the private tertiary care hospital. We used nonprobability consecutive samplings, and the sample size was calculated on Epi Info software (Centers for Disease Control and Prevention, Atlanta, GA) by using a prevalence of 5.3% and the estimated sample size of 215 neonates with a bound of error of 3% with 95% confidence interval [3]. Term neonates (i.e., those born at 37 to 40 weeks plus six days weeks) and late preterm neonates (i.e., those born at 34 weeks to 36 weeks plus six days) of both sexes were included in the study. We excluded preterm neonates (i.e., those born <34 weeks of gestation), post-term neonates (i.e., those born > 42 weeks of gestation), and neonates with sepsis, congenital malformations, congenital heart disease, and any with syndromes such as Down syndrome or Edward syndrome.

After approval from the Ethical Review Committee, all subjects fulfilling the eligibility criteria were enrolled after providing informed verbal and written consent from the mother or father. Neonates born late preterm and term were monitored for up to 72 hours. The patient data confidentiality was maintained by assigning a number for each patient's data, electronic data were password-protected, and data on hard copies were kept in locked storage. All study data were stored for five years. After completion of the study, results can be shared with the patient's parents upon request. The primary investigator did not have control over the management of the study patients.

Data were collected on a proforma and included necessary demographic information. Gestational age, birth weight, gender, mode of delivery, and complications such as hypoglycemia and neonatal jaundice were recorded. Data were analyzed using IBM SPSS Statistics for Windows, Version 20.0 (IBM Corp., Armonk, NY). Descriptive statistics were analyzed for demographic, gestational age, birth weight, sex, mode of delivery, neonatal hypoglycemia, and neonatal jaundice. Continuous variables were presented as mean ± standard deviation. Frequency and percentages were calculated for sex, mode of delivery, gestational age category, hypoglycemia, and neonatal hyperbilirubinemia requiring phototherapy. Complications were evaluated for both term and late preterm neonates using the Chi-square test. A p-value of <0.05 was considered significant. Confounders had been restricted in eligibility criteria. All possible confounders such as clinical sepsis and metabolic disorders were filtered at recruitment. However, if any complications occur in recruited infants, those cases were excluded from the analysis.

Stratification to gestational age, weight at birth, sex of the neonate, and mode of delivery was done. Post-stratification chi-square test was applied, and p <0.05 was considered significant.

## Results

**Figure 1:**
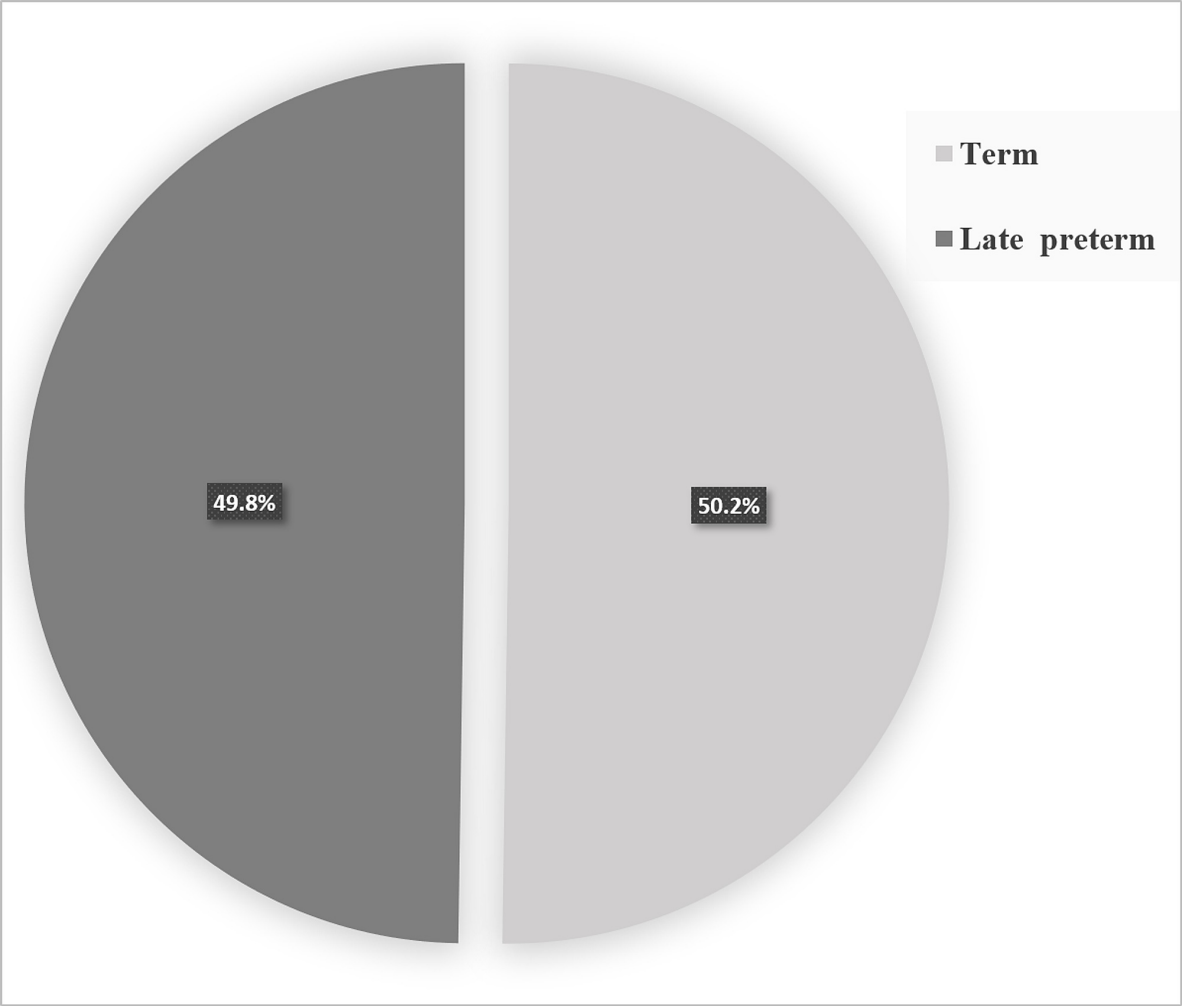
Percentage of gestational age

**Figure 2:**
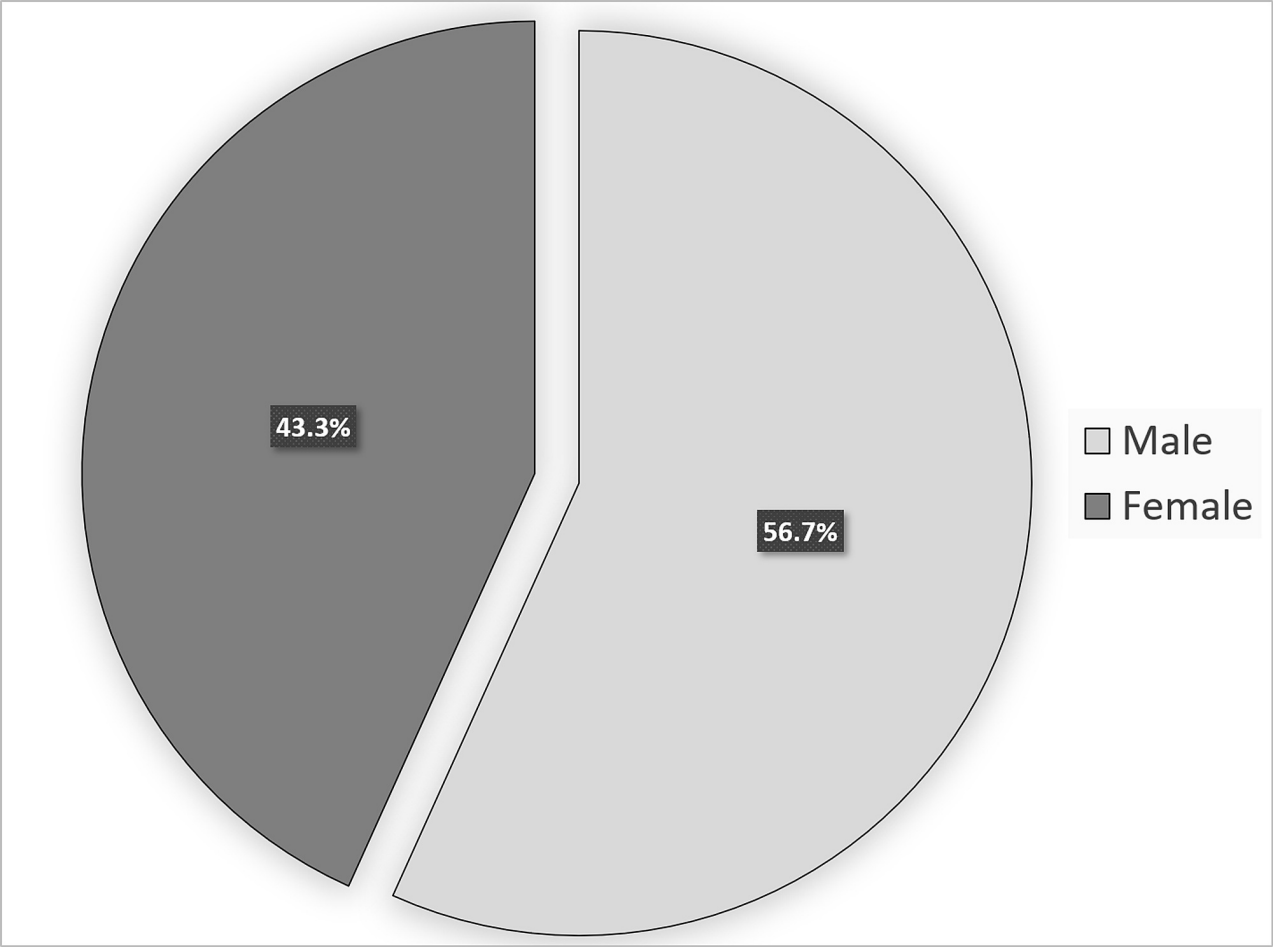
Percentage of gender

**Figure 3:**
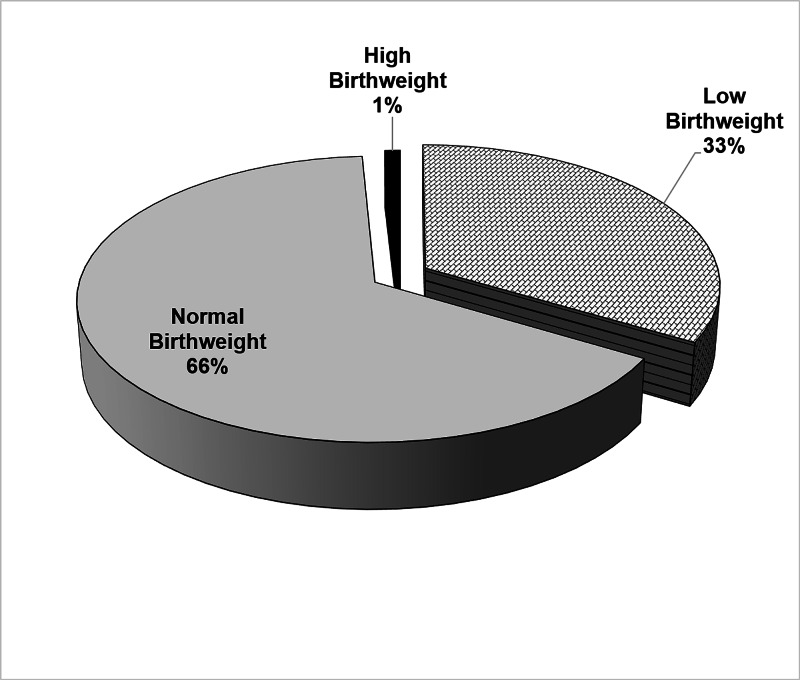
Percentage of birth weight category

A total of 215 neonates were born during the study period, of whom 108 (50.2%) were term babies and 107 (49.8%) late preterm babies (Figure 1). There were 122 (56.7%) male neonates, and 93 (43.3%), female neonates, as shown in Figure 2. The mean gestational age was 36.90 ± 1.76 weeks (range, 33 to 38 weeks), and the mean birth weight of the enrolled neonates was 2.710 kg ± 0.526 kg (range, 1.54 to 4.2 kg; Table 1). We found that 33% of the population had low birthweight, 66% had healthy birthweight, and 1% had high birth weight (Figure 3).

**Table 1:** Descriptive statistics

Variables	Mean	Std. Deviation	95% Confidence Interval for Mean
Gestational age (weeks)	36.90	1.76	36.66
Birth weight (kg)	2.71	0.526	2.640

**Figure 4:**
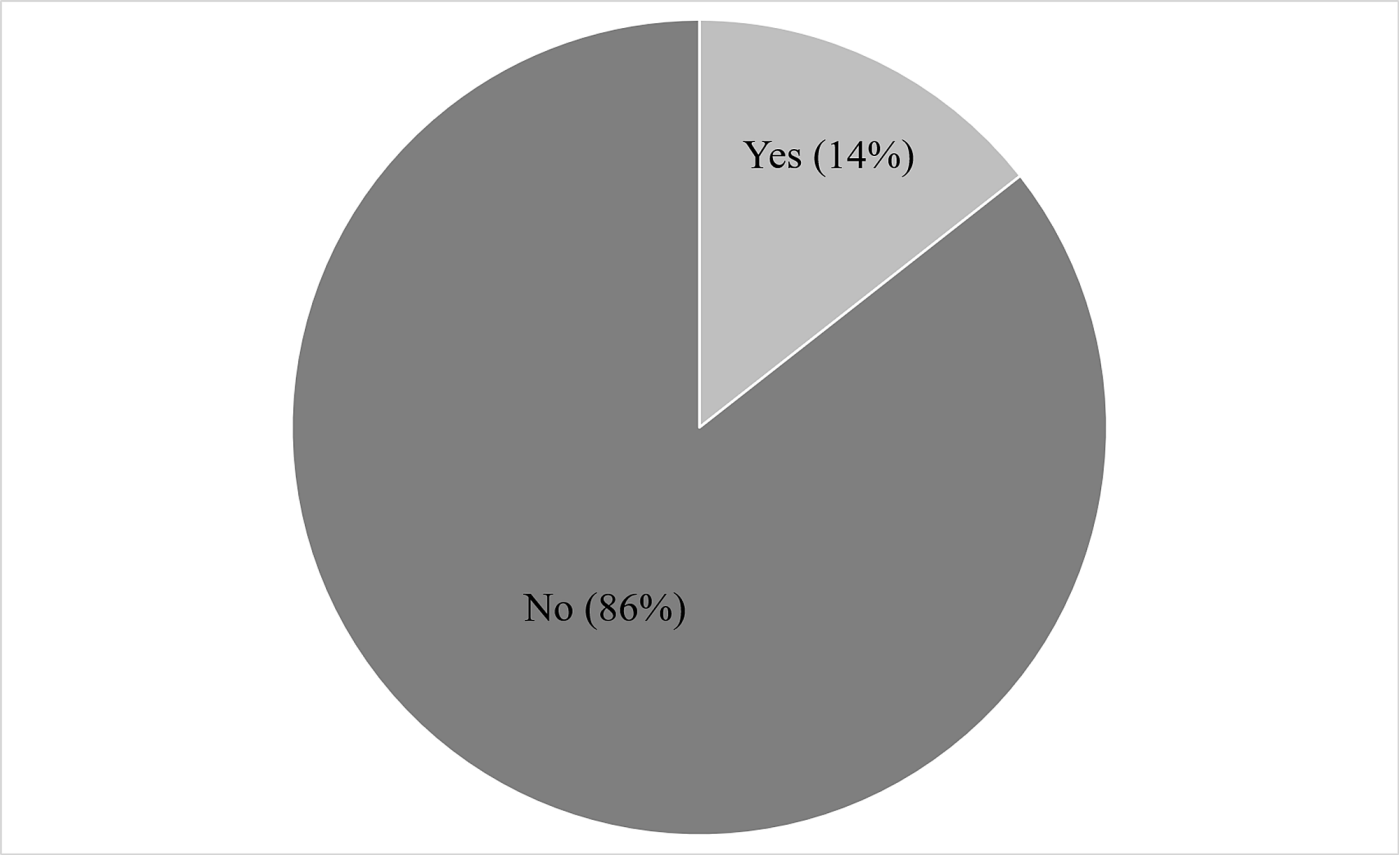
Frequency of jaundice

**Figure 5:**
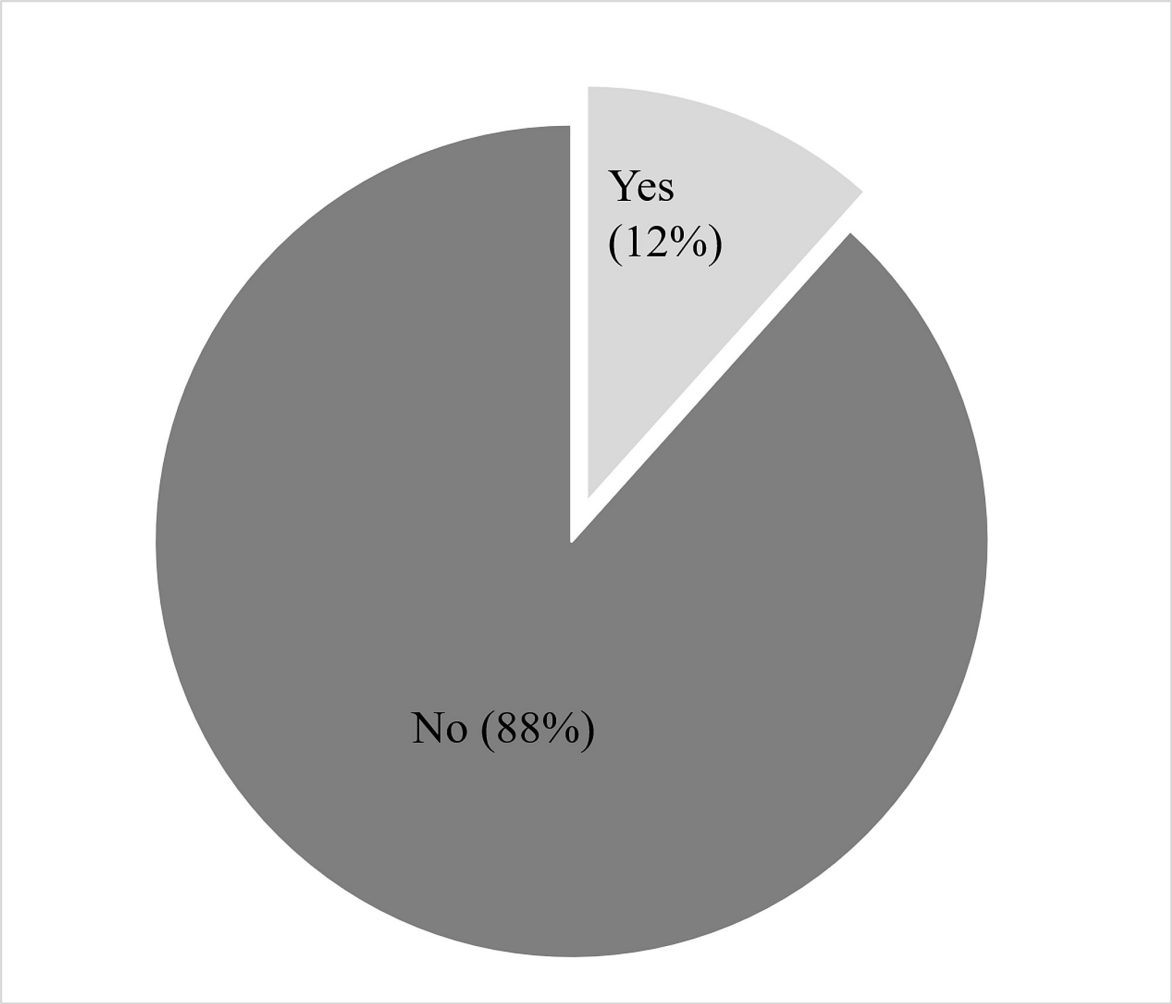
Frequency of hypoglycemia

One hundred fifty neonates (69.8%) were delivered by cesarean section while 65 (30.2%) were delivered by spontaneous vaginal delivery (Table 2). Jaundice was noted in 31 (14%) neonates (Figure 4), and hypoglycemia was noted in 25 (12%) neonates (Figure 5).

**Table 2:** Frequency of mode of delivery C-section: cesarean section; SVD: spontaneous vaginal delivery

Mode of Delivery	Frequency	Percentage
C-section	150	69.8%
SVD	65	30.2%

There is a significant association between gestational age and neonatal complications like jaundice and hypoglycemia. Jaundice was observed 6.5% (n=7) of term neonates and 22.4% (n=24) of late preterm neonates (p<0.01). Similarly, hypoglycemia was observed in only 4.6% (n=5) of term neonates and 15.9% (n=17) of late preterm neonates (p<0.01).

We found no sex predominance for any complications (p>0.05), nor did we find an association between complications and mode of delivery (p>0.05). However, complications declined with increased birth weight, but the association was not statistically significant (p>0.05; Table 3).

**Table 3:** Associations of age, sex, delivery, and birth weight SVD: spontaneous vaginal delivery; C-section: cesarean section; LBW: low birth weight; NBW: normal birth weight/birth weight within reference range; HBW: high birth weight

Variable		Jaundice, n (%)	p-value	Hypoglycemia, n (%)	P-value
Age					
	Term	7 (6.5%)	<0.01	5 (4.6%)	<0.01
	Late preterm	24 (22.4%)		17 (15.9%)	
Sex					
	Male	13 (10.7%)	0.07	12 (9.8%)	0.825
	Female	18 (19.4%)		10 (10.8%)	
Delivery mode					
	SVD	7 (10.8%)	0.316	4 (6.2%)	0.194
	C-section	24 (16%)		18 (12%)	
Birth weight					
	LBW	14 (19.4%)	0.294	12 (16.7%)	0.083
	NBW	17 (12.1%)		10 (7.1%)	
	HBW	0		0	

## Discussion

Compared with term neonates, neonates born late preterm (34 weeks to 36 weeks) are at higher risk for early morbidities. Several important differences in clinical outcomes appear when comparing the late-term neonates' hospital courses with term neonates. Despite their appropriate size and favorable Apgar scores, late preterm infants had significant complications such as jaundice and hypoglycemia when compared with term neonates. A study conducted in the Division of Neonatology at Hacettepe University in Turkey from 2001 and 2002 evaluated 365 neonates (219 term and 146 late preterm neonates) for jaundice [7]. Twenty-three term (10.5%) and 37 late preterms (25.3%) neonates had significant jaundice, which was similar to our results. Sarici et al. reported that late preterm neonates had significantly lower birth weights, higher serum total bilirubin levels, and were 2.4 times more likely to develop significant hyperbilirubinemia than term neonates [7]. Similarly, we found that complications decline with the increase in birth weight among the neonates, but the difference was not statistically significant (p>0.05).

Jaiswal et al. reported that 70.8% of late preterm neonates and 29.1% of term neonates had at least one neonatal morbidity like neonatal jaundice, hypoglycemia, respiratory morbidities, and sepsis that may need observation in hospital [5]. They observed jaundice in 55.1% of late preterm neonates who required phototherapy, and hypoglycemia was found in 8.8% of late preterm neonates. Only 24.8% of term neonates had jaundice, and 1.4% had hypoglycemia [5]. In results similar to our findings, Jaiswal et al. reported that, compared with term infants, late preterm infants were at higher risk for jaundice and hypoglycemia.

Wang et al. noted significant differences in the rate of hypoglycemia for the term (5.3%) and late preterm (15.6%) infants (p=0.028) [3]. Our study produced similar results with 4.6% of term neonates and 15.9% in late preterm neonates experiencing hypoglycemia (p<0.01).

Our study was limited in that we were unable to assess feeding difficulty and breastfeeding status due to hospital policy to supplement feeding to all neonates. Also, the study did not address other early complications and long-term complications. Finally, the study used a relatively short monitoring period, and we did not conduct a long-term follow-up to monitor outcomes.

## Conclusions

There is a significant association between gestational age and immediate neonatal complications of jaundice and hypoglycemia. Compared with term neonates, late preterm neonates are at a higher risk of neonatal jaundice and hypoglycemia, and gender and mode of delivery did not correlate to complications rate. Therefore, to prevent early neonatal jaundice and hypoglycemia, efforts should encourage proper gestation age, good birth weight, and proper antenatal care to reduce neonatal mortality and morbidity. Strong communication between patients, hospital staff, and obstetricians can help ensure the proper time of induction of labor and Caesarean section and improve care at birth and afterward for optimal outcomes for all neonates.
